# Effects of two training workshops upon university students’ learning of critical thinking

**DOI:** 10.1371/journal.pone.0316760

**Published:** 2025-01-06

**Authors:** Carlos Ossa Cornejo, Silva F. Rivas, Carlos Saiz Sánchez

**Affiliations:** 1 Educational science department, University of Bío-Bío, Concepción, Chile; 2 Basic psychology, psychobiology and methodology of behavioral sciences, University of Salamanca, Salamanca, Spain; University of the Basque Country UPV / EHU, SPAIN

## Abstract

We evaluated the effect of two workshops on learning about critical thinking amongst first- year university students. 66 Chilean pedagogy students participated, all female, with ages ranging between 18 and 37 years. Two experimental groups were organized, one with direct teaching (n = 22) and another one with semi-directed teaching (n = 19), plus a control group (n = 25). Pre- and post-tests were applied with an abbreviated Pencrisal critical thinking questionnaire. The results showed statistically significant differences in both experimental groups between the pre- and post-test, with a greater difference in experimental group 1, while the control showed no significant differences. The covariables evaluated were age and academic performance upon entering university, observing that even after controlling for the aforementioned variables, there are differences between the experimental groups and the control. We discuss the possible positive effect of direct teaching in critical thinking as a more effective strategy.

## Introduction

Critical thinking is a highly valued and in-demand skill in our time, since it is seen as a cognitive tool useful for facing the challenges of current education. In fact, it is incorporated as part of the thinking skills for the 21st century, together with creativity and problem-solving [1]. These cognitive processes are becoming necessary skills for adequate social performance [2]. It has been considered a necessary element for developing skills and abilities which can help improve learning quality, and ultimately student performance, in order to develop their autonomy and self-efficiency [3]. A critical thinker can achieve higher levels of reflection, certainty, and depth in their knowledge [4].

These characteristics make critical thinking a relevant factor for university education, given the potential benefits it can offer regarding the development of scientific knowledge, comprehensive reading strategies, reflective analyses, decision making, etc. [5–8], even when it is not always clear how it can be incorporated into the curricular and didactic structures of university majors [9, 10].

The present study specifically approaches the need to improve the formative process in Initial Teacher Training, particularly for preschool education professionals, who are in charge of supporting the formation of thinking in small children. While these professionals are not expected to incorporate critical thinking skills into their teaching methodologies, it has been said that, given the delicate nature of their work, they should use this type of skill to strengthen their professionalism and decision-making [11].

## Definition of critical thinking

Critical thinking is considered a complex construct, having affinities with some concepts related to reflective and evaluative capacity, including rationality and a critical sense [12]. It differs from them inasmuch as it is a cognitive tool which can strengthen and regulate thought itself as a producer of knowledge, beyond being an epistemological posture about knowledge [13].

It is a way of thinking where the subject improves the quality of the process by taking charge of the inherent structures of the act of thinking and bringing them up to intellectual standards [14]. It is also considered a thinking skill which helps evaluate the merit, precision, and/or authenticity of the information being created or learned; being oriented towards both information and action in a problem-solving context and in interactions with other people [15–17].

It has been stated that it is difficult to achieve precision or consensus on the concept, since there are different definitions and characteristics in its evaluation [12, 18–20]. Two elements align in this. First, that it is a facet of knowledge and human sapience, which has been related with various disciplines and epistemologies. One of these is philosophy, which sees critical thinking as an action or art of questioning the truth, as well as knowledge and its relation with power. Another approach to critical thinking comes from psychology, which sees it as a cognitive mechanism promoting individual development [21, 22].

Second, and more specifically from the psychological perspective, there are different models aiming to explain how and in which ways cognitive processes can explain the appearance of this skill. Some establish the supremacy of analytical processes, while others indicate the existence of arguing as a process leading to analyses, and others even seek to balance the emergence of analytical and argumentative processes with metacognition [18, 23–25]. Despite these divergences, its cognitive and reflective essence is recognized as a preponderant factor [13].

However, there has been a consideration for various years that critical thinking is not only a cognitive process, and is instead closely tied to motivational factors (or personal disposition) which are needed for its proper development [12, 26]. These cognitive and motivational skills which form the grounds for critical thinking are also complemented by metacognition, which is the capacity for self-monitoring and evaluation of thoughts and actions [27].

From an educational perspective, critical thinking is considered as a set of complex skills developed in a sociocultural training space, focused on presenting macro-skills which guide the analysis and use of information, relating it with problem solving and decision making, where specific skills are subprocesses which report information to the two more global processes [3]. This focus is presented as a more social and contextualized perspective, since its focus is to endow people with an effective tool for adapting to their needs. On the other side, it can provide better orientation to developing tasks related with didactic and curricular activities in university training as currently suggested.

### Types of critical thinking training

There are some experiences in development of critical thinking which are centered on adapting the techniques and skills of critical thinking to instructional strategies, in order to be able to work on them in the classroom via curricular insertion. López [15] states that both writing tasks and instructor feedback can positively affect the development of students’ critical thinking skills. Assigning students tasks based on independent research projects, group work projects, presenting to the class group, and essay-based tests also appear to develop critical thinking.

Other programs have been developed which do not have curricular insertion and which are organized in particular activities, specifically aimed at developing the skills less related with didactic situations where logic predominates, and more related with problem-solving and decision-making situations, which are not common subjects in educational curricula [10, 24].

There are various proposals and focuses where we can see the work of promoting critical thinking in education:

- Direct teaching models: a more traditional focus from psychology, centered on identifying specific cognitive skills or focusing on thinking, which seeks to develop strategies promoting a reflective process about ideas which can improve comprehension. These include the models from Lipman (philosophy for children), Toulmin (arguing-centered), or Project Zero (based on ideas from Goodman y Perkins) [24].
- ABP-based models: These state that one way to develop significant learning and critical thinking arises in situations which problematize the student, motivating them to present their own solutions which must be justified based upon arguments and evidence. One experience from this focus is SPRI (Situation, Problem, Resolution, Information) from Parra & Lago [28]; another one is the ASARPIC model (Significant Learning via Solving an Integrative and Contextualized Problem) from Sánchez [29]. There is also the ARDESOS model ARDESOS (Argumentation, Decision, Solving problems in daily Situations) developed by Saiz & Rivas [30].
- Thinking infusion models: These are framed by the so-called Specific domain focus, which proposes that it is not possible to work on thinking habits in a general way and decontextualized from a knowledge area such as science or politics. The infusion model is one way to include thinking skills in the topics of a knowledge area, but with direct and explicit instructions for its development [31].
- Models based upon reading activities and critical thinking: Critical thinking is worked on via reading activities, and abased on critical questions which can be done individually or in pairs [32, 33].
- Scientific reasoning models: These proposals work on reasoning skills, based on heuristics and logic, to establish adjustment criteria regarding information and determining conclusions towards which the data or cases analyzed can be guided [34].

### Objective

The study objective is to analyze the effect of two programs on critical thinking (on one side, from a direct teaching model, and on the other, from an infusion model) in pedagogy students at a university to see their effectiveness and explore differences in their results.

## Method

A quasi-experimental study was done with two experimental groups and a control group, applying a pre- and post-test.

### Participants

The participants were 66 university students in the first year of the preschool education major, all women, with ages ranging from 18 to 37 years (M = 20.02, S.D. = 2.64). There were 3 study groups, one experimental (Exp 1) with direct teaching (n = 22), another experimental group (Exp 2) with semi-directed teaching (n = 19), and a control group (n = 25) without any critical thinking training, but which did have critical analysis and reasoning activities included in their courses.

### Instruments

To evaluate critical thinking, we used the abbreviated critical thinking questionnaire (PENCRISAL), consisting of 6 items and two dimensions. It is based upon open-response questions rated on the basis of performance criteria. The original instrument presented adequate reliability levels, with a Cronbach’s α between 0.63 and 0.74 [35, 36], and the abbreviated version presenting a model with good indicators of fit [37].

For covariable values, we also obtained data from the students which appear on their institutional files, specifically age and their academic performance level as measured by the national evaluation applied to all students entering university education in Chile, currently called the Higher Education Access Test or PAES [38]. This is an evaluation which measures knowledge and skills learned in the areas of mathematics, language, history, and science. To measure performance levels in basic educational areas, we use the average for language and mathematics, with a value between 100 and 1000 points.

### Procedures

The research project underwent an evaluation by the bioethics committee in the institution, and a written informed consent was applied before collecting the data. We began by contacting the head of the major in order to coordinate data gathering actions and workshop implementation. The students chosen for the intervention were in their first year in the major, and were divided randomly into two experimental groups via a raffle system. The control group is the immediately previous cohort, which is in their second year.

Experimental group 1 underwent a workshop for critical thinking training with direct teaching, via modeling and self-instruction exercises. 4 sessions of 80 minutes each were done once per week. Experimental group 2 had a semi-directed training workshop, with examples and self-instruction exercises, but where instead of modelling the skill, moments for reflection and dialogue on the activities were incorporated. The control group had no interventions in developing critical thinking.

The three groups were evaluated one week before, and one week after having finished the experimental group workshops. Data of pretest was obtained between October 03 and October 10, 2023; Data of postest was obtained between November 21 and November 27,

2023.

For data analysis we used descriptive statistics, with an analysis of central trend measurements, dispersion, and distribution, as well as inferential (parametric) statistics, applying a Student T-test with repeated measures for pre- and post-test group comparison, along with a covariance analysis to evaluate the influence of the age and academic performance variables on the result of learning about critical thinking. Normality and homoskedasticity assumptions were evaluated via a Kolmogorov-Smirnov (K-S) test and a Levene test.

## Results

The results obtained indicate that the averages obtained in the groups between the pre- and post-test present differences (see Table 1).

**Table 1:** Values for mean differences between pre- and post-test.

Group	Mpre (S.D.)	Mpost (S.D.)	t	p
Experimental 1	2.45 (2.08)	5.00 (2.50)	-4.254	0.0001
Experimental 2	2.21 (2.15)	4.46 (2.80)	-2.249	0.037
Control	3.44 (1.66)	2.76 (1.92)	1.48	0.153

In the pre-test application, we can observe that the control group had greater results than both experimental groups, which may be due to acquired thinking and academic arguing habits gained at university. The experimental groups had similar values in the pre-test, with a slightly larger but non-significant difference in group 1.

**Fig 1:**
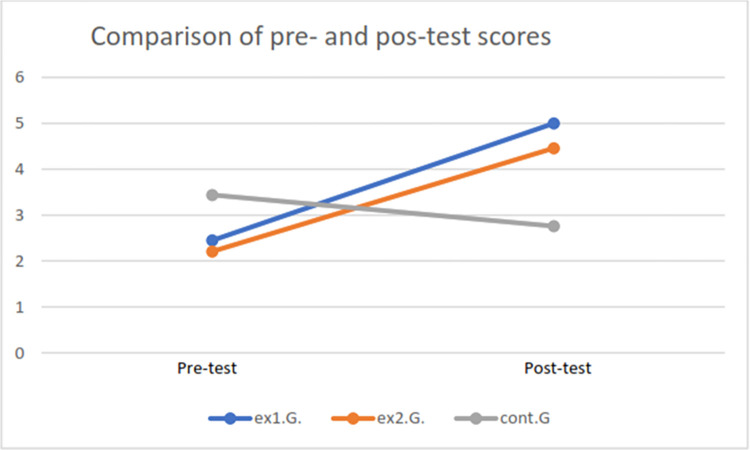
Graph of results from groups in pre- and post-test evaluations.

For the post-test, we can see that experimental group 1 (ex1.G.), which had a workshop based on direct teaching with exercises modeled on critical thinking skills (arguing and decision- making), presented higher values in the post-test than the other two groups. Similarly, experimental group 2 (ex2.G.), which underwent a semi-directed teaching workshop, had a higher level than in the pre-test as well as being higher than the control group (control.G.). In the post-test, the control group had a lower mean than in the pre-test, possibly because the importance required for carrying out the evaluation was not taken, although this difference is not statistically significant. Fig 1 presents this in graphic form.

We note that there are statistically significant differences between the two experimental groups in the evaluations before and after the intervention, with an increase between the two evaluations, while in the control group, although there is a negative difference, this is not statistically significant.

Since there could be different reasons for the effect of the workshop in achieving critical thinking, such as age or academic performance, which could influence its outcomes, we applied a covariance analysis to determine these covariables’ possible influence.

Basic analyses were applied to evaluate the statistical assumptions of the covariance test for the dependent variable (post-test score), with normality appearing in the sample (K-S > 0.05), homoskedasticity (Levene > 0.05), normality of residuals (K-S > 0.05) and variable independence. We can observe that age does not present a statistically significant relation with post-test critical thinking scores (r = 0.106, p<0.1), while academic performance does have one, but at a medium-low level (r = 0.230, p<0.05).

Table 2 shows that the covariables’ values, once controlled, generate no influence on the intervention modality used to achieve critical thinking amongst the students. We can also note that the adjusted R2 would explain only 16% of the variance, which is considered unimportant.

**Table 2:** ANCOVA values’ relation with academic performance and age values as covariables.

Origin	Type III of squares’ sum	gl	Quadratic mean	F	Sig.
Corrected model	98.154ª	4	24.539	4.169	0.005
Intersection	29.394	1	29.394	4.994	0.029
Age	2.144	1	2.144	0.364	0.548
PAES/PSU	15.210	1	15.210	2.584	0.113
Group	78.830	2	39.415	6.697	0.002
Error	359.012	61	5.885		
Total	1427.000	66			
Total corrected	457.167	65			

a. R squared = 0.215 (R2 adjusted = 0.163)

Table 3 also indicated statistically significant differences as a function of the group types (workshops), with a large size effect as a function of the Cohen values for F-tests [39].

**Table 3:** ANOVA values on post-test scores.

Variable	M (DE)	M (DE)	M (DE)	F	p	η2*	CI1	CI
	Exp_1	Exp_2	Control				inf.	sup.
Post-test	5.00	4.46	2.76	6.977	0.002	0.181	0.030	0.328
Critical	(2.50)	(2.80)	(1.92)					
Thought								

* Eta squared (η2)

1 CI = confidence Interval at 95%

Finally, we can indicate that the post-hoc Bonferroni test also showed that the groups’ difference did not emerge between the experimental groups themselves, but between the two experimental groups and the control.

## Discussion

Based on the data obtained, we can determine that there are differences in the results for the effects on each experimental group between the initial and final evaluation, although the data indicate that group 1 has a clearer effect than group 2. We can also observe that the control group had no differences between the pre- and post-tests. The difference in results in the pre- test in favor of the control group may be due to the participants’ training level, since the control group members are in their second year, while both experimental groups’ participants were first-year students.

The post-test also shows that while both experimental group students increased their performance, the control group participants saw theirs fall, aligning with another study with a similar group of participants [40]. This may be because the control group does not establish a motivation for carrying out the test, since they see no benefit or result from it, a situation which even though it is not optimal for the study, does not generate problems as long as the difference is statistically insignificant [41].

Regarding the results from the two workshop types in the experimental groups, we found significant differences between the pre- and post-tests, with greater significance in experimental group 1 which worked with a methodology based on direct instruction, compared to experimental group 2 which used a semi-directed methodology with direct instructions and reflective and inquiring strategies. This post-test difference could be due to the need to train thinking to achieve an adequate level of critical thought, which cannot be easily achieved naturally [24].

Direct instruction has had good results as a methodology for promoting critical thinking by offering reasoning models and examples about how to carry out reasoning actions [42]. However, these differences may be due to the participants’ cognitive traits as well, since in the post-test analysis, no statistically significant differences were found between the two experimental groups, since the reflection and inquiry methodology is equally useful for developing critical thinking [32, 43].

The preceding point could thus also be confirmed by controlling for variables including age and academic performance, although differences persist in groups’ performances. The covariance analysis lets us control the influence of possible variables which emerge along with the dependent variable in an experiment [44]. Certain mentions have been made of critical thinking being variable with the age and maturity levels of people [45–48], although there is only statistical data for the relation between critical thinking and academic performance. The fact that the differences continue with the covariable control allows us to bet on the effectiveness of the methodologies carried out in the workshops for critical thinking development, more than for individual traits.

Finally, we can present the need to continue studying how to promote critical thinking skills when training future educators, since it is an important aspect in the training process, as reported by various studies analyzing the inclusion of this skill in educational processes [49]. This is particularly relevant for early education workers, since it can lay the grounds to boost thinking from early human development stages [50].

## Conclusions

The promotion of critical thinking in teacher training is a relevant topic today, with much research in the health and education fields. However, there are not always reports of studies with experimental (or quasi-experimental) methodology amongst university students, perhaps due to its complexity. Our study data can make a positive contribution about incorporating methodologies to promote critical thinking which can be incorporated into education on an intra-curricular basis, and validate direct instruction as a viable strategy. However, we should note that this requires proper understanding of the strategies for the explanation and modelling to be effective.

There is also a need to go into depth about the possible existence of differences in achieving critical thinking as a function of methodologies, since our results were nuclear as to whether one of the strategies could be more effective than the other for promoting this skill. It would also be interesting to see whether there are differences with other methodologies such as those based on ABP, which have also shown important effects on developing critical thinking [51, 52].

The limitations of the study include the unrepresentatively small sample size, which keeps us from making generalizations about the effectiveness of the methodologies considered, along with the use of intact groups. To continue strengthening the study, it needs to be replicated in other groups of preschool educators training in higher education institutions with similar characteristics, and using randomly constituted groups.

Another element which may have limited achieving other results was the limitation in sessions, which is considered low according to the standard achieved in other studies which have worked on this skill, and which have run between 6 and 16 sessions [41, 53, 54]. While significant differences were found, which shows the strength of the methodology, it may only be a sample of the mechanical learning of the skill, which can be lost over time or not be transferred to other life areas, both of which are crucial for achieving significant learning for this skill [55].

Finally, we consider it necessary to continue developing and testing methodologies to promote critical thinking in higher education, particularly in educators’ initial training, since it is an area which has seen little work to date, due to the issues with working on this skill, evaluating it, and integrating it into educators’ professional performance profiles. It is a challenge which can be overcome to the extent that more studies show positive experiences in such situations.
